# Discovery of Sphingosine-1-Phosphate Receptor Modulators as Potential CHI3L1 Inhibitors by Ligand-Based Virtual Screening and Molecular Dynamics Simulations

**DOI:** 10.1021/acsomega.5c01968

**Published:** 2025-05-06

**Authors:** Elnaz Aledavood, Carmen Gil, Manuel Comabella, Ana Martinez

**Affiliations:** 1 Centro de Investigaciones Biologicas “Margarita Salas” (CIB-CSIC), Ramiro de Maeztu 9, Madrid 28040, Spain; 2 Servei de Neurologia. Centre d’Esclerosi Múltiple de Catalunya (Cemcat). Institut de Recerca Vall d’Hebron (VHIR), Passeig de la Vall d’Hebron 129, Barcelona 08035, Spain; 3 Centro de Investigación Biomédica en Red en Enfermedades Neurodegenerativas (CIBERNED), Instituto de Salud Carlos III, Melchor Fernández Almagro 3, Madrid 28029, Spain

## Abstract

Multiple sclerosis is characterized by central nervous system inflammation, demyelination, and neuronal degeneration. Current diagnostic and prognostic methods lack precision, necessitating biomarkers for personalized treatment strategies. Chitinase 3-like 1 (CHI3L1) has emerged as a potential prognostic marker, with elevated levels correlating with disease severity and relapse risk. Despite its therapeutic potential, few CHI3L1 inhibitors have been identified. Using ligand-based virtual screening and molecular dynamics simulations, Food and Drug Administration-approved drugs have been screened as CHI3L1 inhibitors with the final goals of being repurposed in MS and other inflammatory diseases, offering promising therapeutic approaches. This investigation suggests that sphingosine-1-phosphate receptor modulators such as fingolimod could be potential inhibitors for CHI3L1.

## Graphical abstract

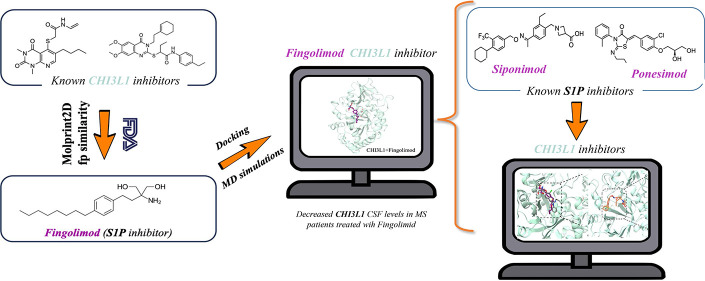

## Introduction

Multiple sclerosis (MS) is an autoimmune disorder affecting the central nervous system (CNS), causing chronic inflammation, demyelination, and neuronal degeneration. Diagnosis of MS relies on identifying characteristic lesions in at least two distinct CNS areas.^1^ Recent studies have shifted focus toward atrophy and cortical lesions over lesion quantity and relapse rates for predicting disease progression and cognitive impairment in MS. However, current assessment methods, mostly subjective, often lack precision, highlighting the need for personalized treatment strategies based on individual prognosis and risk assessment.^2^ Biomarkers play a crucial role in predicting disability progression, monitoring disease activity, and assessing treatment response in MS. Biomarkers are measurable characteristics indicating normal biological processes, disease development, or response to treatment. In MS, biomarkers aid in early diagnosis, improving patient care, and disease management.^3^ Among various cerebrospinal fluid (CSF)/serum biomarkers, Chitinase 3-like 1 (CHI3L1), also known as YKL-40, has recently emerged as a potential biomarker for MS.^4^ The expression of CHI3L1 has been found to be higher in the CSF of individuals with MS.^5^

CHI3L1, a glycoprotein expressed in various tissues and cells such as macrophages, neutrophils, chondrocytes, and endothelial cells, plays a role in inflammation, tissue injury, repair, and remodeling across different tissues.^6^ Studies have shown that elevated levels of CHI3L1 in CSF are associated with a greater likelihood of conversion from clinically isolated syndrome (CIS-MS) to clinically definite MS.^7^ Additionally, higher levels of CHI3L1 in CSF are correlated with increased disability in MS patients.^8^ Also, individuals with higher concentrations of CHI3L1 in CSF have a significantly heightened risk of relapse, increased MRI activity, and evidence of disease activity.^9^

In humans, CHI3L1 has a molecular weight of 40 kDa and is encoded by the *CHI3L1* gene. Belonging to the chitolectin class of glycosyl hydrolase family 18, this protein bears significant homology to chitotriosidase (CHIT1) and acidic mammalian Chitinase.^10^ However, a mutation at a crucial residue Glu140 to Leu140 renders it enzymatically inactive, and as a results, CHI3L1 lacks chitin hydrolase activity.^11^ Despite this, they maintain the ability to bind to chitin oligomers. While CHI3L1 has emerged as a target in various clinical studies, there is a limited number of documented inhibitors with confirmed affinity for CHI3L1. Only a couple of small molecules have been discovered and used successfully as CHI3L1 inhibitors in different preclinical models. CHI3L1 binders can inhibit the protein through various mechanisms of action. They may interfere with CHI3L1-receptor interactions, such as those with IL-13Rα2, either by disrupting glycosaminoglycan-mediated binding or through direct interaction with the receptor. Another mode of action involves small-molecule inhibitors that block the release of cytokines by CHI3L1 from the extracellular matrix.

In the recent last years, structure-based drug discovery has led to the identification of some inhibitors targeting CHI3L1 (Figure 1). In 2021, Lee et al. discovered that the binding of K284 to the chitin-binding domain (CBD) of CHI3L1 inhibited its interaction with the interleukin-13 receptor subunit alpha-2 (IL-13Rα2), effectively suppressing the signal mediated by CHI3L1.^12^ Subsequently, in 2022, another inhibitor of CHI3L1, G721-0282, demonstrated antianxiety effects by blocking CHI3L1-mediated neuroinflammation.^13^ More recently, and during the preparation of this manuscript, two compounds (including compound 30) exhibiting nanomolar affinity for CHI3L1 have been identified, and the structure of CHI3L1 bound to these compounds has been successfully determined (inhibitor 30).^14^ As of now, there has not been any clinically established drug known for its inhibitory effect against CHI3L1 that could be used in patients, including those with conditions like MS, as an effective therapeutic strategy.

**Figure 1:**
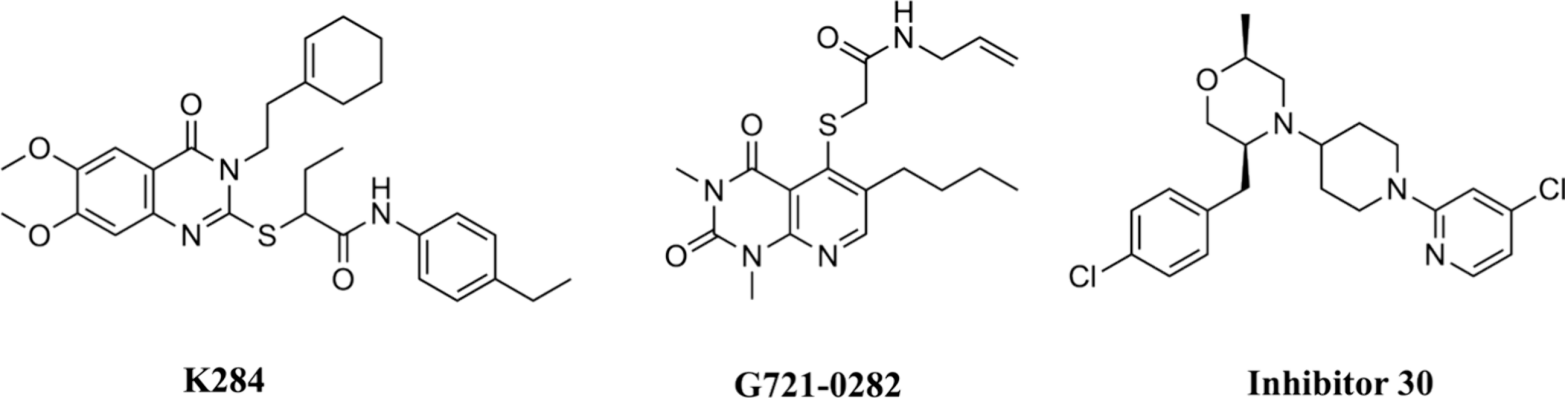
Chemical structures of known CHI3L1 inhibitors.

## Methods

### Ligand-Based Virtual Screening

The FDA-approved drugs have been screened to discover new compounds capable of inhibiting CHI3L1. Four commonly used 2D similarity metrics, namely, Buser, Cosine, Kulczynski, and Tanimoto, have been employed to prioritize potential hits.^15^ This prioritization was based on the linear fingerprint method implemented within Schrödinger’s similarity-based virtual screening protocol.^16^ MolPrint2D fingerprints,^17^ which encode atom environment descriptors based on molecular connectivity tables, were utilized as 2D similarity descriptors. Two CHI3L1 selective inhibitors, G721-0282 ^13^ and K284 ^12^ were chosen as reference structures for the 2D similarity search.

### Docking of the Selected Compounds

The crystal structure of CHI3L1 bound to inhibitor 30 PDB ID: 8R4X^14^ was used for docking the selected compounds. This structure was selected due to being the only X-ray structure available bound to an inhibitor and featuring a high resolution of 1.54 Å. The molecular docking calculations and preparation of both protein and ligands were conducted by using the Schrödinger software package. The protein was prepared and minimized using the Protein Preparation Wizard,^18^ while the chemical structure of selected compounds was optimized by LigPrep.^16^ The compound’s binding modes were determined through GLIDE docking of each compound onto the minimized X-ray structure. The grid box was positioned at the inhibitor binding site, utilizing default parameters for receptor grid generation.^19^ The ligands were docked using GLIDE extra precision (XP) and Induced-fit docking (IFD).^20^

### Molecular Dynamics Simulations

Extended molecular dynamics (MD) simulations were employed to validate the stability of the docking poses and investigate the structural and dynamic features of the simulated systems. To achieve this, the CHI3L1 complexes were constructed using the protein complexed with fingolimod, siponimod, ponesimod, G721-0282, and K284 selected based on the most favorable docking scores. Additionally, to facilitate comparison, the CHI3L1 complexed with compound 30 ^14^ was also simulated to assess its binding stability.

Simulations were conducted using the AMBER20 ^21^ package and Amber ff14SB force field^22^ for the protein, while the ligands (fingolimod, inhibitor 30, G721-0282, K284 siponimod, and ponesimod) were parametrized with the general amber force field (GAFF)^23^ and restrained electrostatic potential (RESP) partial atomic charges^24^ derived from B3LYP/6-31G­(d) calculations. The ionizable residues were assigned their standard protonation states at physiological pH, and ACE and NME capping groups were added to the protein’s N- and C-termini. The systems were embedded in an octahedral box filled with TIP3P water molecules,^25^ and counterions were included to ensure system neutrality. The final setup consisted of the CHI3L1 protein, the ligand, approximately 11,000–12,000 water molecules, and a variable number of Cl ions, resulting in simulated systems containing around 40,000 atoms. Simulations were carried out in the NPT ensemble for equilibration and in the NVT ensemble for molecular dynamics (MD) production, utilizing periodic boundary conditions and Ewald sums (with a grid spacing of 1 Å) to handle long-range electrostatic interactions. All complexes underwent duplicate simulations.

Initially, the systems were minimized by refining the positions of hydrogen atoms in the protein (2000 cycles of steepest descent followed by 8000 cycles of conjugate gradient), then minimizing the water molecules (using the same approach), and finally minimizing the entire system (4000 steepest descent cycles and 1000 conjugate gradient cycles). Subsequently, the system temperature was gradually increased from 100 to 300 K over five steps, each lasting 50 ps, using the NVT ensemble. During this phase, restraints (8 kcal mol^–1^ Å^–2^) were applied to keep the ligand within the binding pocket and prevent artificial rearrangements during equilibration.^26,27^ An additional 5 ns step in the NPT ensemble was performed to equilibrate the system’s density, with the restraints being gradually released in the following steps.^27^ Production MD simulations were conducted for 500 ns per replica, totaling 5 μs of simulation time for the ligand-bound CHI3L1 complexes.

### Essential Dynamics (ED)

This approach was employed to identify the key motions associated with structural variations observed during the MD simulations. ED analysis involves examining and visualizing movements along individual modes separately, facilitating the characterization of the dominant collective motions in the simulations. The method relies on constructing and diagonalizing a positional covariance matrix to extract the collective deformation modes (eigenvectors), with the eigenvalues reflecting the relative contribution of each motion to the overall structural variability of the protein. In this study, ED analysis was applied to 25,000 snapshots obtained from 500 ns of simulation for each system, focusing exclusively on backbone atoms. The computations were carried out using the PCAsuite software (available at http://www.mmb.irbbarcelona.org/software/pcasuite/pcasuite.html) and integrated into the pyPCcazip tool suite.^28^

### MM/GBSA Calculations

The MM/GBSA method designed to evaluate the free energy difference between two states, typically representing the bound and unbound forms of solvated molecules.^29^ It can also be applied to compare the free energies of different solvated conformations of a single molecule. In this study, we employed MM/GBSA scripts integrated within Amber and AmberTools to automate the binding free energy calculations for the protein–ligand complex.

## Results

### Ligand-Based Virtual Screening

In the present study, we performed ligand-based virtual screening (LBVS) to investigate the potential of repurposing Food and Drug Administration (FDA)-approved drugs as inhibitors against CHI3L1. Employing the known CHI3L1 inhibitors, K284 and G721-0282, as scaffolds, our objective is to identify novel compounds capable of inhibiting CHI3L1 that can be repurposed in the clinical setting, thereby offering promising therapeutic approaches for inflammatory diseases like MS.

The main goal of the ligand-based virtual screening (LBVS) was to identify potential novel inhibitors of CHI3L1 by screening FDA-approved drugs chemical library through computational methodologies.^30^ LBVS relies on the concept that compounds with similar structures tend to exhibit similar biological activities. The main ligand-based methodologies involve the use of pharmacophores (abstract representations of the essential features required for a molecule to exhibit activity), shape-based similarity, fingerprint similarity, and employing machine learning techniques using molecular properties and data derived from the aforementioned methods.^31^ In this context, aiming to repurpose FDA-approved drugs, a virtual screening of this library has been conducted to identify novel compounds potentially active against CHI3L1 using 2D similarity metrics, namely, Buser, Cosine, Kulczynski, and Tanimoto. They have been employed to prioritize potential hits.^15^ MolPrint2D fingerprints^17^ were utilized as 2D similarity descriptors. Two CHI3L1 selective inhibitors, G721-0282 ^13^ and K284,^12^ (Figure 1) were chosen as reference structures for the 2D similarity search.

Using fingerprint similarity analysis, a selection was made, identifying a list of approved drugs with similarity indexes exceeding 0.85 in relation to both K284 and G721-0282 (Tables S1 and S2 in the Supporting Information). This list encompasses various drug families, including kinase inhibitors like cabozantinib, alpha-1 adrenergic antagonists such as terazosin and dixazosin, and anti-inflammatory medications such as nabumetone and phenylbutazone. Notably, fingolimod, a chemically diverse compound with an inhibitory effect against sphingosine-1-phosphate receptor (S1P), which has been approved for the treatment of MS, is also present among these drugs.^32^ Its similarity index and chemical structure are reported in Table 1. Fingolimod was selected due to its fingerprint similarity to that of G721-0282. Interestingly, a recent paper describes the reduction of CHI3L1 levels in CSF of MS patients treated with fingolimod, postulating that this protein is an efficacy biomarker related with the inflammation decrease produced by the drug treatment.^33^ These findings are aligned with the outcomes from the LBVS conducted in this study.

**Table 1:**
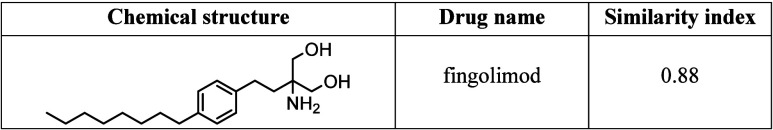
2D Similarity Index (Buser Matrix) of Fingolimod Relative to G721-0282, alongside Its Chemical Structure

### Molecular Docking and Dynamics Simulations

Docking studies were conducted to thoroughly understand the binding mode and inhibition mechanism of fingolimod and the reference compounds G721-0282 and K284. The crystal structure of CHI3L1 (PDB 8R4X) served as the basis for docking the selected compound. The most favorable conformation revealed that fingolimod binds to the pocket similarly to the known inhibitor described in the X-ray structure (inhibitor 30), with a docking score of −10.349, slightly higher in comparison to −9.764 for the inhibitor 30 (Figure 2, orange stick). In terms of inhibitor 30, it establishes hydrogen bonds with Tyr206 and Asp207, along with hydrophobic interactions with Trp352, Thr293, and Phe261.

**Figure 2:**
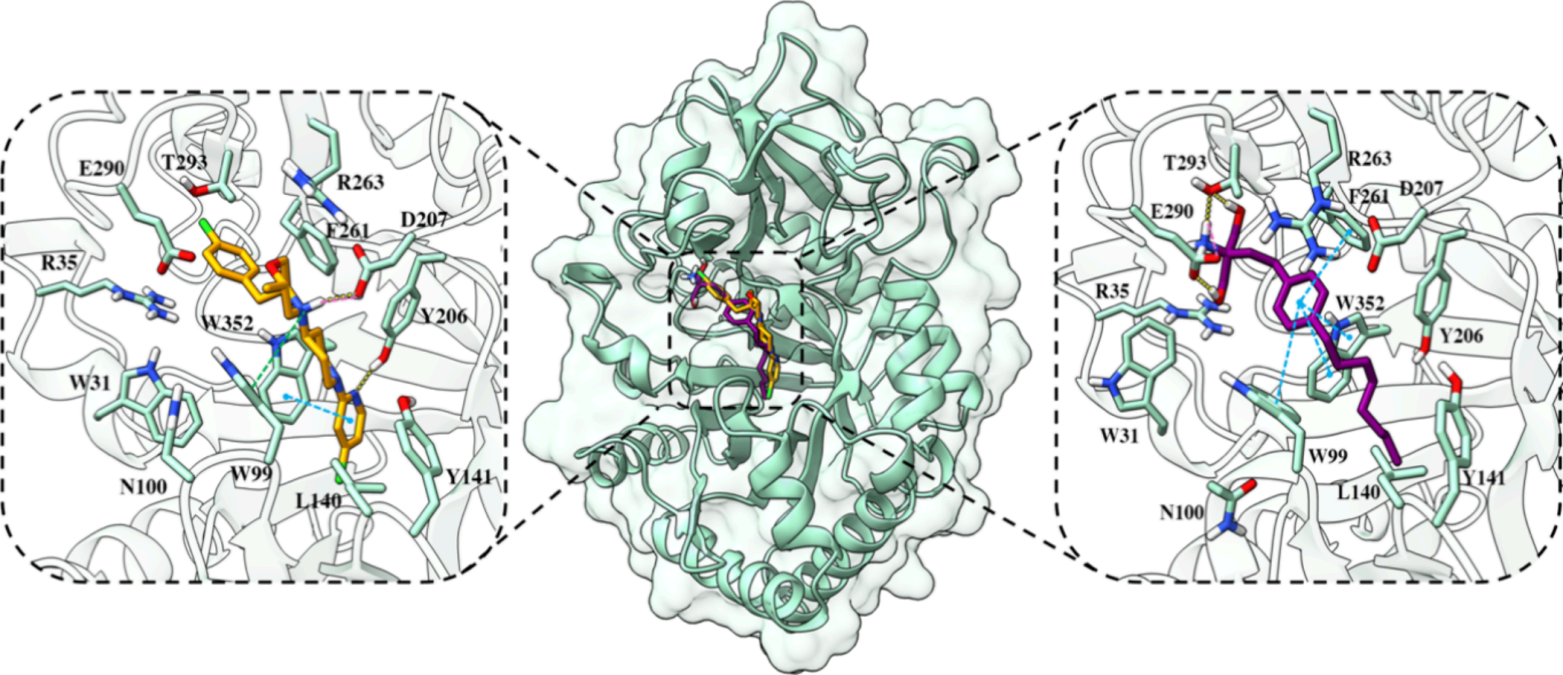
Fingolimod’s binding mode as identified from docking studies and MD simulations (rendered in violet sticks) and inhibitor 30 from the X-ray structure PDB ID 8R4X (rendered in orange sticks) within the cavity (illustrated as a seafoam green cartoon). Detailed close-up of the ligand binding pocket highlights essential residues within a 5 Å proximity of the ligand. The yellow dashed lines denote hydrogen bonds, while the pink dashed lines indicate salt bridges, the blue dashed lines represent π–π stacking, and green dashed lines show π–cation interactions. Two-dimensional diagrams of protein-fingolimod interactions are presented in Figure S2A in the Supporting Information.

Fingolimod is deeply accommodated within the hydrophobic groove, with binding interactions involving hydrogen bonds to Glu290 and Thr293, along with multiple π–π stacking interactions with Trp99, Phe261, and Trp352 (Figure 2, violet stick). The reference compound, G721-0282, is deeply embedded within the hydrophobic groove, where it interacts through π–π stacking with Trp99 and Trp352 and forms a hydrogen bond interaction with N100 (Figure S1A in the Supporting Information). These interactions are thought to be essential for the inhibition of CHI3L1, as previous studies indicate that the compound effectively disrupts the movement of the flexible Trp99 residue.^14^ In addition, K284 also engages the hydrophobic groove through π–π-stacking interactions with Trp99 and Trp352, reinforcing its stable positioning within the binding site. Beyond these aromatic contacts, K284 forms several hydrogen bonds with key polar and charged residues including Arg35, Arg293, and Glu290, all of which are located within the CBD of CHI3L1 (Figure S1B in the Supporting Information).

Fingolimod is approved for the treatment of MS based on its mechanism of action: modulation of the sphingosine-1-phosphate receptor (S1P). Additionally, two more drugs, namely, siponimod^34,32^ and ponesimod^35^ (Figure 3), chemically diverse to fingolimod but with documented efficiency against S1P, have been also recently approved for MS clinical treatment.

**Figure 3:**
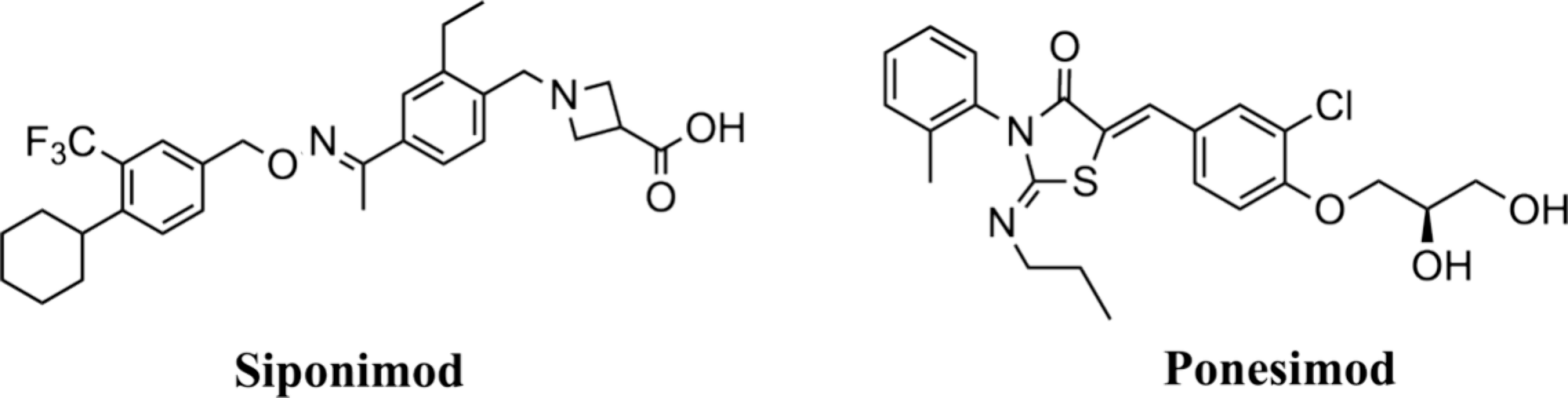
Chemical structures of the known S1P inhibitors.

Here, we selected these two compounds to assess their ability to bind to the hydrophobic pocket of CHI3L1. The research aims to investigate if they are CHI3L1 binders and if they interact with key protein residues, as observed in the binding of inhibitor 30 and fingolimod.

Ponesimod exhibits docking scores of −11.076 and binds to the CHI3L1 binding site similar to fingolimod and inhibitor 30. It forms hydrogen bonds with Arg35 and Thr293 and interacts with the backbone nitrogen atom of Tyr99, alongside a π–π stacking interaction with the same residue, resembling fingolimod’s behavior (Figure 4, shown as gray stick).

**Figure 4:**
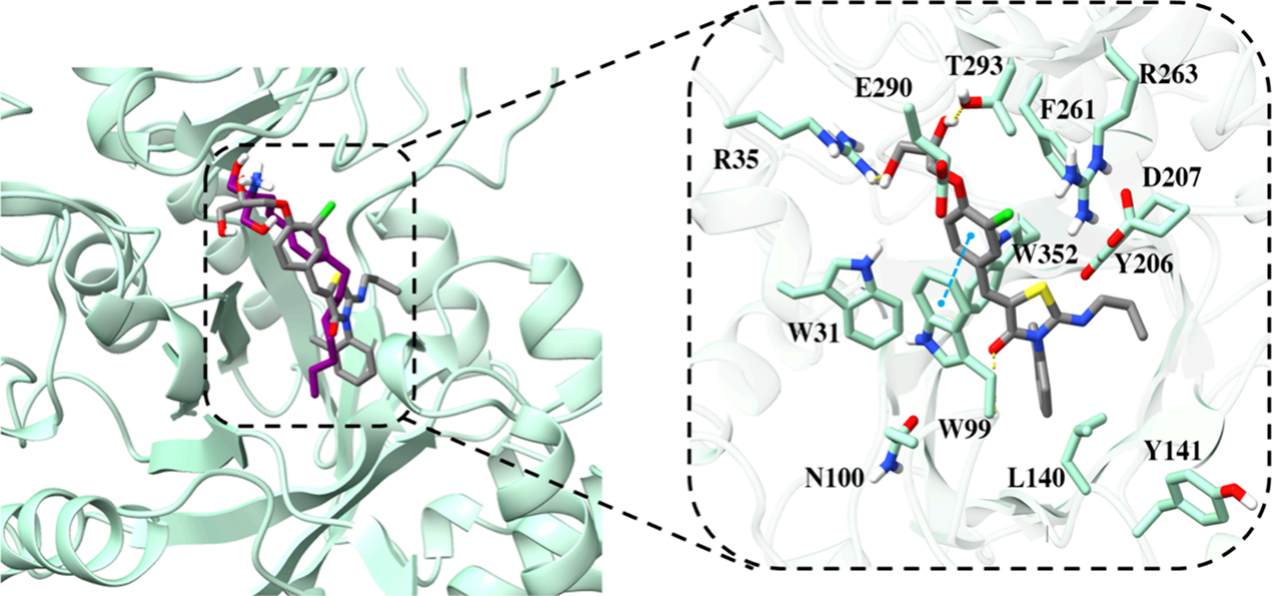
Superposition of fingolimod and ponesimod’s binding modes (rendered in violet and gray, respectively) as identified from MD simulations within the cavity (illustrated as a seafoam green cartoon). Detailed close-up of the ponesimod’s binding pocket, highlighting essential residues within a 5 Å proximity of the ligands. The yellow dashed lines denote hydrogen bonds, while the pink dashed lines indicate salt bridges, the blue dashed lines represent π–π stacking, and green dashed lines show π–cation interactions. Two-dimensional diagrams of protein–ponesimod interactions are presented in Figure S2B in the Supporting Information.

Compared with the S1P inhibitors mentioned earlier, siponimod displays a distinct binding pattern with a docking score of −12.941. It establishes salt bridge and hydrogen bond interactions with Asp207. Additionally, it exhibits π–π stacking interactions with Trp31, as well as a cation−π interaction with Tyr352 (Figure 5, depicted in salmon stick). Notably, siponimod’s binding mode is distinguished by forming both the salt bridge and hydrogen bond interactions with the same residue as inhibitor 30, a feature solely observed in siponimod’s case.

**Figure 5:**
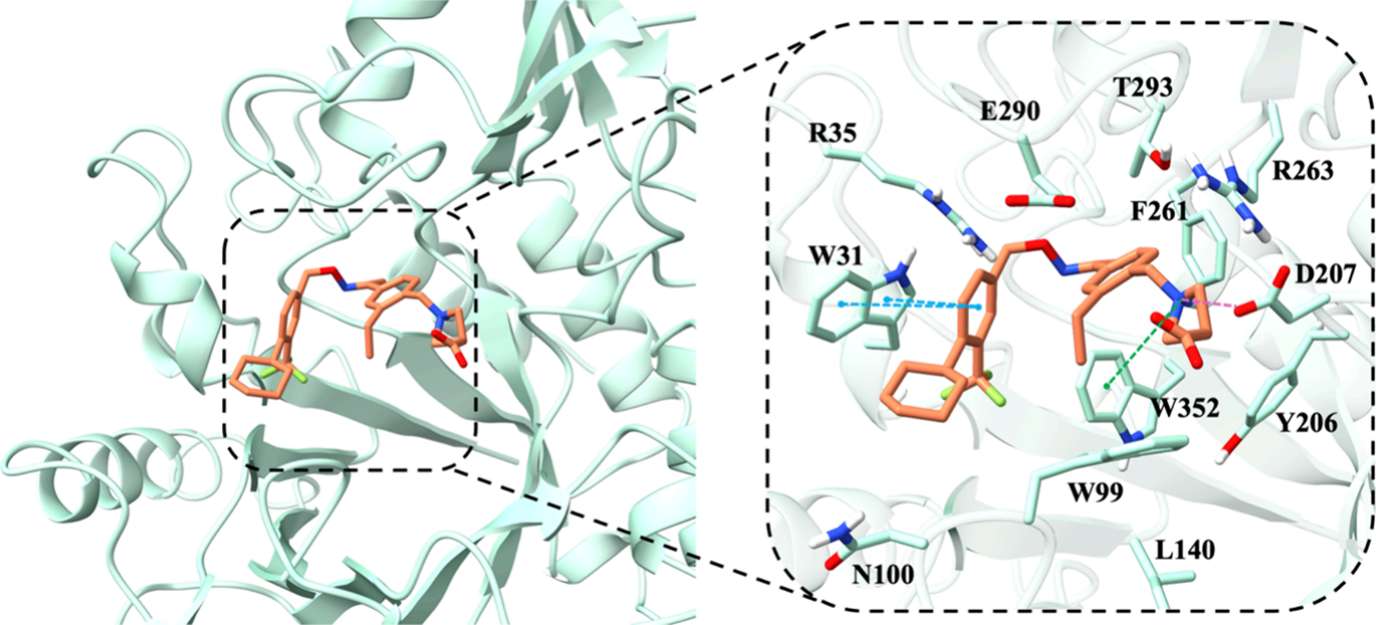
Siponimod’s binding mode as identified from MD simulations (rendered in salmon stick) within the cavity (illustrated as a seafoam green cartoon). Detailed close-up of the ligand binding pocket, highlighting essential residues within a 5 Å proximity of the ligand. The pink dashed line indicates salt bridge, the blue dashed lines represent π–π stacking, and the green dashed line shows π–cation interactions. Two-dimensional diagrams of protein-siponimod interactions are presented in Figure S2C in the Supporting Information.

### Structural Analysis of CHI3L1 Complexes

CHI3L1 inhibition can be achieved by binding inhibitors to its oligomer binding pocket. Small molecules that fit into the hydrophobic groove may either directly block the enzyme or influence, through allosteric mechanisms, the interaction between the CHI3L1 complex and other proteins such as IL-13Rα2. To observe the protein’s dynamic response to inhibitor binding, we performed multiple molecular dynamics (MD) simulations, analyzing trajectory files by measuring the root-mean-square deviation (RMSD) and root-mean-square fluctuation (RMSF) of the protein backbone throughout the simulations. The RMSD analysis of CHI3L1 protein complexes with different compounds over a 500 ns MD simulation reveals a generally consistent stability pattern across all systems (Figure 6). All of the complexes exhibit RMSD values that stabilize below 2 Å, indicating stable protein–ligand interactions and minimal structural deviation over time. While minor fluctuations are observed, particularly in the CHI3L1-siponimod complex, these variations do not suggest significant instability but rather reflect the dynamic nature of molecular interactions. Overall, the results demonstrate comparable binding behaviors across all of the compounds, reinforcing their potential to form reliable and stable interactions with CHI3L1. This consistency underscores the suitability of these ligands for further analysis and potential application.

**Figure 6:**
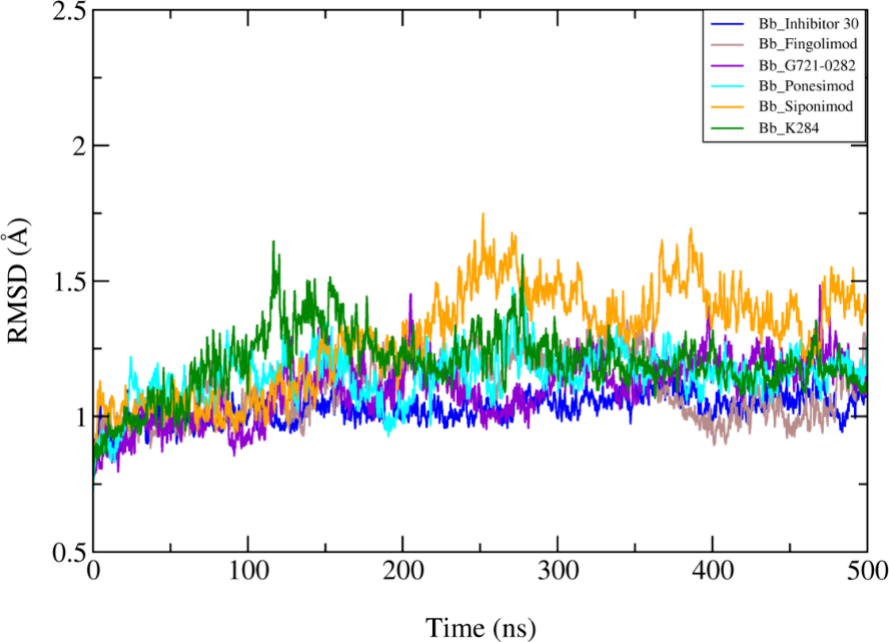
RMSD of CHI3L1 protein complexes with different compounds in 500 ns MD simulations. The RMSD values for inhibitor 30 (blue), fingolimod (brown), G721-0282 (violet), ponesimod (cyan), siponimod (orange), and K284 (green) indicate stable protein–ligand interactions, with all complexes stabilizing below 2 Å.

The RMSF analysis indicates that the complexes display comparable fluctuation profiles with siponimod and ponesimod exhibiting the highest levels of flexibility. While most residues exhibit fluctuations below 2Å, specific regions, including residues 200–220, 230–242, and 261–281, show increased fluctuations, suggesting greater flexibility in these segments (Figure 7A). To further elucidate the effects of inhibitor binding on CHI3L1 dynamics, ED analysis was performed to assess the influence of ligand binding on the principal motions of the protein backbone. The ED results revealed that inhibitor binding induces conformational changes in CHI3L1 complexes. Among the studied inhibitors, inhibitor 30 exhibited the lowest backbone motion, suggesting that its binding reduces protein flexibility, thereby indicating its potential as a potent inhibitor. In contrast, as observed in the RMSF analysis, other ligands, such as siponimod, demonstrated flexible regions corresponding to the CBD (Figure 7B, orange: CHI3L1-siponimod complex; blue: CHI3L1-Inhibitor 30 complex), implying comparatively lower stability of these ligands within the binding site (the ED analyses of fingolimod, G721-0282, K284, and ponesimod, along with the contributions of the essential motions, are presented in Figure S3 and Table S3 in the Supporting Information).

**Figure 7:**
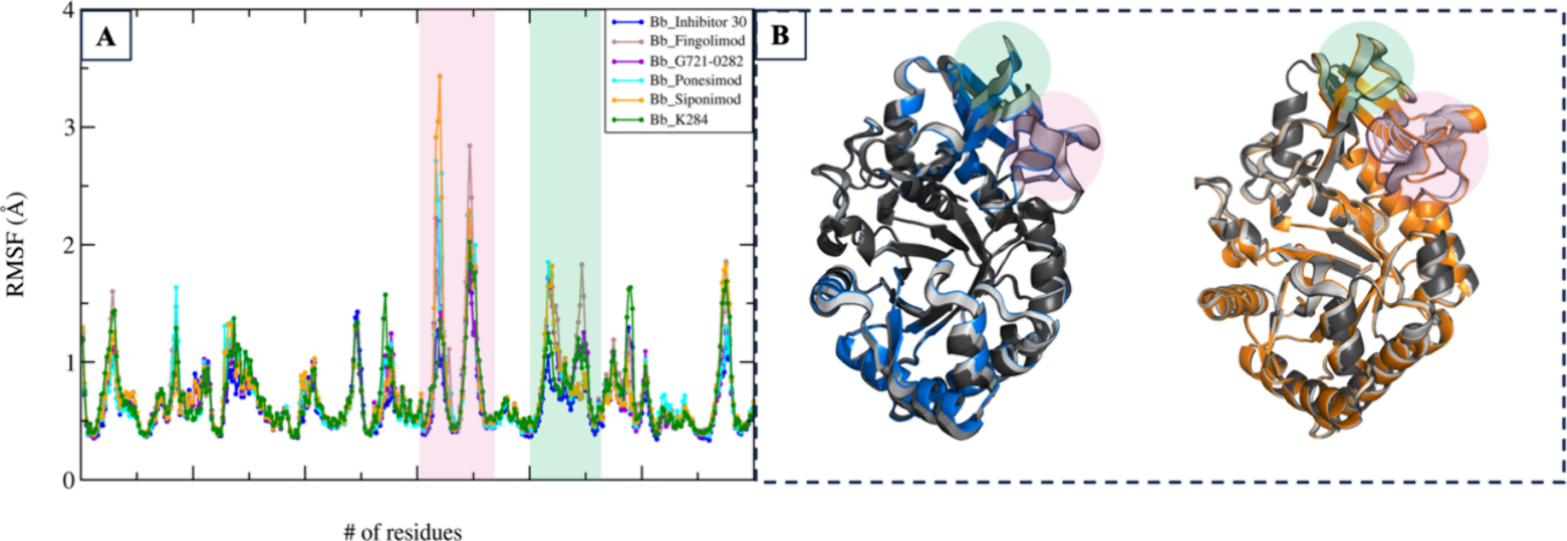
(A) RMSF (Å) averaged across two independent replicas for all simulated systems with highlighted regions corresponding to the CBD. (B) ED analysis of 500 ns MD simulations for CHI3L1-siponimod and CHI3L1-inhibitor 30, represented in orange and blue, respectively. The first principal motion of the Cα atoms is depicted.

The MM/GBSA binding free energy results for six compounds—inhibitor 30, fingolimod, G721-0282, K284, ponesimod, and siponimod—bound to CHI3L1 are displayed in Figure 8. Among the compounds, inhibitor 30 demonstrates the most stable interaction, with a binding free energy of −53.9 kcal/mol, suggesting its high affinity for the binding site. Fingolimod follows as the second most stable ligand, with a binding free energy of −44.2 kcal/mol. G721-0282, K284, and ponesimod show slightly lower stabilities, with values of −40.1, −42.3, and −43 kcal/mol, respectively. Siponimod exhibits the least stability, with a binding free energy of −36.2 kcal/mol.

**Figure 8:**
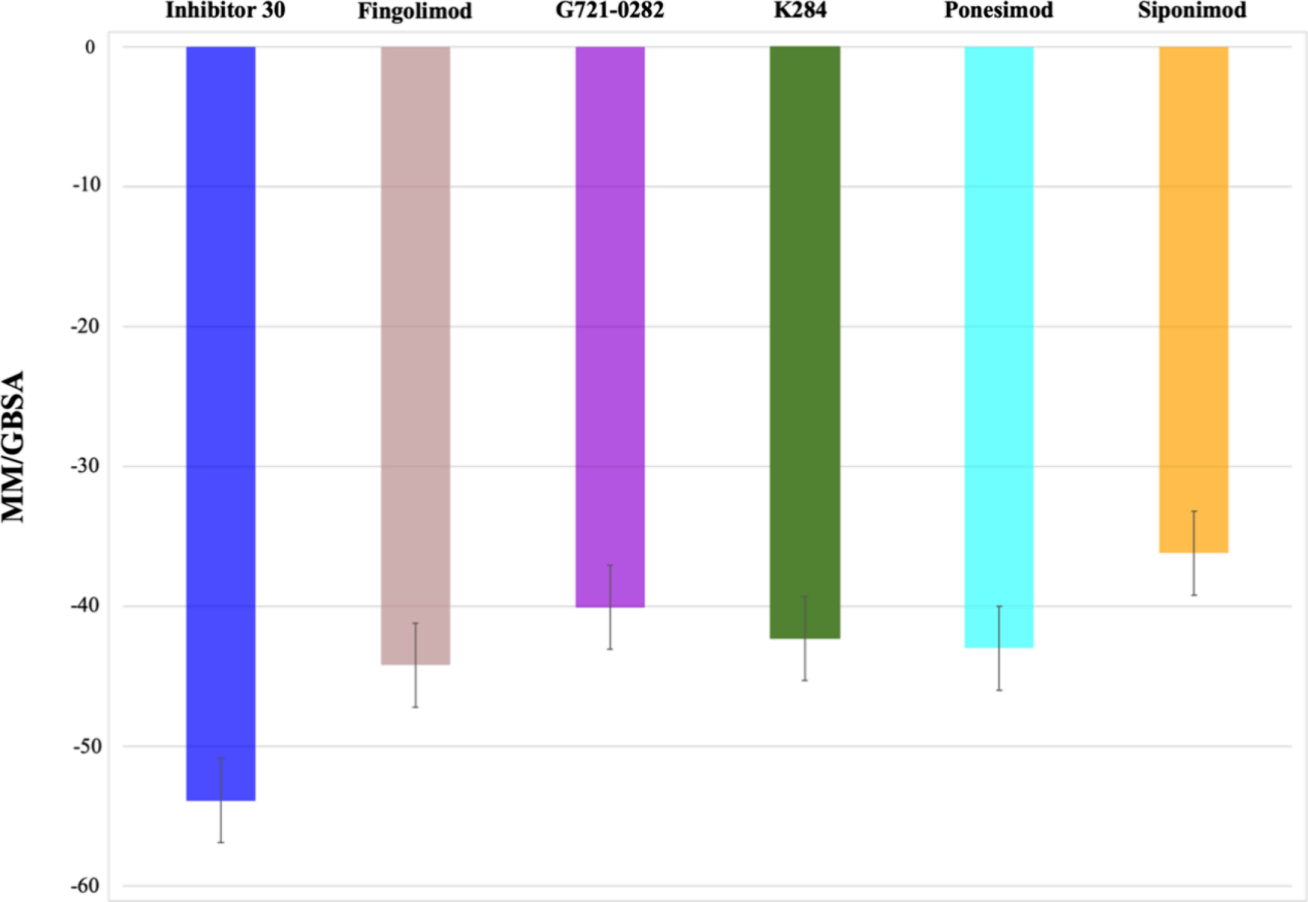
MM-GBSA binding free energy results for the six compounds—inhibitor 30 (blue), fingolimod (brown), G721-0282 (violet), K284 (green), ponesimod (cyan), and siponimod (orange)—bound to the CHI3L1 protein.

These results are consistent with previous analyses, which further highlight the stabilizing effects of inhibitor 30 and fingolimod on the protein–ligand complex. The agreement between MM/GBSA and other structural analysis findings emphasizes the reliability of the computational simulations, confirming that inhibitor 30 is the most stable ligand, followed by fingolimod and ponesimod.

## Discussion and Conclusions

Multiple sclerosis is a chronic and multifactorial disease characterized by inflammation and demyelination in the central nervous system. Its complex pathophysiology and heterogeneous clinical presentations require the development of innovative therapeutic strategies that can address the diverse needs of patients.^2^ Biomarkers have become essential in advancing MS diagnostics, improving patient classification, guiding therapeutic decisions, and refining disease management. Among these, Chitinase-3-like protein 1 has emerged as a particularly promising prognostic biomarker, highlighting its potential as a therapeutic target in MS.^4^ Its upregulation in MS patients, along with its reduction following treatment, supports its active involvement in disease mechanisms rather than its being a passive marker. CHI3L1 plays a role in driving neuroinflammation, glial activation, and tissue remodeling, key processes in MS pathology. Targeting CHI3L1 aims to disrupt these pathways by inhibiting its interaction with receptors, such as IL-13Rα2, thereby reducing downstream inflammatory signaling. Although CHI3L1 inhibitors may not directly suppress its expression, they block its function and may help interrupt the feedback mechanisms that sustain its elevated levels.

However, despite the increasing interest in CHI3L1, there is a notable lack of small-molecule inhibitors with a confirmed affinity for this protein, with only a limited number reported in preclinical models. This gap underscores the need for further research and development of novel CHI3L1 inhibitors. This study aimed to address this challenge by employing ligand-based virtual screening^30^ to identify potential inhibitors of CHI3L1 from a library of FDA-approved drugs. This approach leverages the safety profiles and established pharmacokinetics of existing drugs to streamline the drug discovery process, bypassing the lengthy and expensive stages of de novo compound development. Using fingerprint similarity methods and two selective CHI3L1 inhibitors, G721-0282 ^13^ and K284,^12^ as reference structures for our 2D similarity search, we identified fingolimod, an FDA-approved sphingosine-1-phosphate (S1P) receptor modulator, as a potential CHI3L1 inhibitor. Notably, clinical evidence suggests that fingolimod treatment reduces CHI3L1 levels in the cerebrospinal fluid of MS patients,^33^ further validating its relevance as a candidate for CHI3L1-targeted therapy.

Although fingolimod is known to exert its approved pharmacological action via its phosphorylated form, previous pharmacokinetic studies have shown that both phosphorylated and nonphosphorylated forms coexist in the blood.^36,37^ These findings support the plausibility that the parent compound may be available at sufficient levels in vivo—particularly in the CNS—to interact with CHI3L1, as suggested by our computational results.

To expand on these findings, two additional S1P modulators, siponimod and ponesimod, were included in molecular docking and molecular dynamics (MD) simulations alongside fingolimod and a reference CHI3L1 inhibitor (inhibitor 30) derived from the X-ray crystal structure of CHI3L1 (PDB ID: 8R4X).^14^

MD simulations provided critical insights into the stability and dynamics of the protein–ligand interactions over a 500 ns time scale, supporting the outcomes of the docking studies. Inhibitor 30 exhibited the most stable binding profile, maintaining strong and persistent interactions with key residues, such as Trp99, Trp352, and Trp31 within the hydrophobic groove of CHI3L1. These interactions, including hydrogen bonds, salt bridges, and stacking interactions, constrained the local conformational mobility of the protein, as evidenced by the reduced root RMSF values for these residues. Fingolimod and ponesimod showed intermediate stability, forming frequent interactions with key binding site residues, which aligned with the docking predictions. Although their RMSD traces were slightly higher than those of inhibitor 30, they demonstrated stable binding conformations that effectively restricted the global protein motions, as shown by the ED analysis.

Siponimod displayed a distinct binding orientation compared with the other compounds. While its RMSD values were slightly higher, indicating greater conformational flexibility in the binding pocket, siponimod consistently formed critical interactions with different residues, such as Asp207. These interactions align with those observed in the cocrystallized CHI3L1–inhibitor complex, suggesting that siponimod’s binding may require subtle conformational adjustments to achieve optimal stability. Despite its unique binding profile, siponimod’s ability to interact with essential residues highlights its potential as a CHI3L1 inhibitor, warranting further investigation. Binding free energy calculations further validated these findings, ranking inhibitor 30 as the most stable binder, followed by fingolimod, ponesimod, G721-282, K284, and siponimod. These results underscore the consistency between docking predictions and MD simulation outcomes, reinforcing the potential of repurposing S1P modulators for CHI3L1-targeted therapy in MS. Importantly, our findings also highlight the ability of the CHI3L1 binding pocket to accommodate ligands with varying protonation states and charge distributions. Positively charged molecules such as fingolimod and inhibitor 30 engaged the protein through salt bridges and hydrogen bonds with complementary charged and polar residues. In contrast, neutral compounds like ponesimod, G721-0282, and K284 adopted different binding strategies, relying primarily on hydrophobic contacts and π–π stacking interactions, particularly with aromatic residues such as Trp99, Trp352, and Trp31 (the superimposed binding modes of inhibitor 30, G721-0282, and fingolimod are shown in Figure S4 of the Supporting Information). The zwitterionic nature of siponimod enabled both electrostatic and polar interactions with residues like Asp207. This diversity in binding modes suggests that CHI3L1 possesses a chemically versatile binding site capable of stabilizing structurally and electrostatically diverse ligands. Such flexibility enhances the feasibility of targeting CHI3L1 with a broader range of small molecules and supports the rationale for ligand-based screening approaches that include chemically diverse scaffolds.

In addition to the S1P receptor modulators, the LBVS approach identified several other FDA-approved drugs with distinct mechanisms of action, including kinase inhibitors, alpha-1 adrenergic antagonists, and anti-inflammatory agents, which shared structural similarities with the reference inhibitors. These findings expand the pool of potential CHI3L1 inhibitors, which presents new opportunities for further exploration. However, additional in vitro and in vivo studies are essential to confirm the binding mechanisms, therapeutic efficacy, and safety profiles of these candidates. Such investigations will provide a foundation for developing novel treatment targeting CHI3L1, addressing the unmet therapeutic needs in MS, and potentially other inflammatory diseases.

In conclusion, this study highlights the potential of repurposing FDA-approved drugs, particularly S1P receptor modulators such as fingolimod, ponesimod, and siponimod, as inhibitors of CHI3L1, a promising therapeutic target in MS. By leveraging ligand-based virtual screening, molecular docking, and molecular dynamics simulations, we identified and validated the stability and binding affinity of these compounds for CHI3L1, with results aligning consistently across computational approaches. Fingolimod’s known ability to reduce CHI3L1 levels in clinical settings further supports its potential utility in modulating CHI3L1-driven inflammatory pathways.

Beyond S1P modulators, our findings suggest a broader chemical landscape of FDA-approved drugs with structural similarities to known CHI3L1 inhibitors, warranting further experimental evaluation. These results not only underscore the value of computational tools in accelerating drug discovery but also open new avenues for therapeutic development targeting CHI3L1, addressing critical unmet needs in MS treatment and potentially benefiting other inflammatory conditions. To strengthen these computational predictions, it is essential to test the identified compounds *in vitro* to confirm their binding to CHI3L1 and to assess their functional inhibitory activity. Such validation will be crucial to establish their therapeutic relevance and support progression toward preclinical development.

## Supplementary Material



## Data Availability

The structures representing the binding mode of all compounds can be found at 10.5281/zenodo.14900502

## Notes

List of FDA-approved drugs selected using fingerprint similarity, along with 2D diagrams of protein–ligand interactions, ED analyses, and SMILE of all the compounds (PDF)
