# Supplementary material for: Discovery of Sphingosine-1-Phosphate Receptor Modulators as Potential CHI3L1 Inhibitors by Ligand-Based Virtual Screening and Molecular Dynamics Simulations
Source: ACS Omega. 2025 May 6;10(19):19992–20000. doi: 10.1021/acsomega.5c01968 (PMC12096210; doi:10.1021/acsomega.5c01968)
Supplement: Supplementary file 1 [file ao5c01968_si_001.pdf]

SUPPORTING INFORMATION

Discovery of Sphingosine-1-Phosphate Receptor Modulators as Potential CHI3L1 Inhibitors by Ligand-Based Virtual Screening and Molecular Dynamics Simulations

Elnaz Aledavood,[1,*] Carmen Gil,[1,3] Manuel Comabella,[2,3] Ana Martinez[1,3,*]

[1]Centro de Investigaciones Biologicas "Margarita Salas" (CIB-CSIC), Ramiro de Maeztu 9, 28040 Madrid, Spain

[2]Servei de Neurologia. Centre d'Esclerosi Múltiple de Catalunya (Cemcat). Institut de Recerca Vall d'Hebron (VHIR), Passeig de la Vall d'Hebron 129, 08035 Barcelona, Spain.

[3]Centro de Investigación Biomédica en Red en Enfermedades Neurodegenerativas (CIBERNED), Instituto de Salud Carlos III, Melchor Fernández Almagro 3, 28029 Madrid, Spain

Table of content

- **Page S2-S4: Table S1.** The list of FDA-approved drugs with similarity index greater than 0.85 to K284
- **Page S5-S7: Table S2.** The list of FDA-approved drugs with similarity index greater than 0.85 to G721-0282
- **Page S8: Figure S1.** Binding mode and two-dimensional diagrams of protein-G721-0282 and protein-K284 interactions
- **Page S9: Figure S2.** Two-dimensional diagrams of protein-fingolimod and protein-ponesimod interactions
- **Page S10: Figure S3.** The ED analyses of fingolimod, G721-0282, K284, and Siponimod
- **Page S11: Figure S4.** Superimposed binding poses of Inhibitor 30, G721-0282, and fingolimod
- **Page S12: Table S3.** Contribution of the essential motion (%) to the structural variance of different CHI3L1 complexes
- **Page S13:** SMILE of the compounds presented in the manuscript

Table S1. The list of FDA-approved drugs with similarity index greater than 0.85 to K284

Drug bank info	Similarity index	Structure
DB08875<br>Cabozantinib: a tyrosine kinase inhibitor used to treat advanced renal cell carcinoma	0.900615	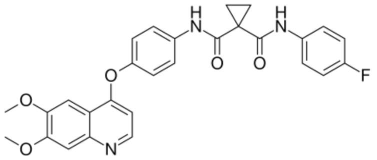
DB06774<br>Capsaicin: a topical analgesic agent used for the symptomatic relief of neuropathic pain	0.877251	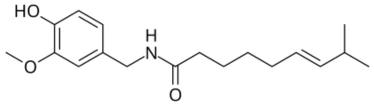
DB09120<br>Zucapsaicin: a topical analgesic used as an adjunct to relieve severe pain of osteoarthritis of the knee	0.877251	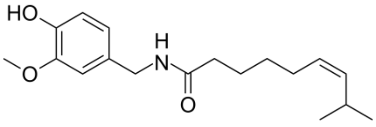
DB02546<br>Vorinostat: a histone deacetylase inhibitor used for the treatment of cutaneous manifestations	0.870529	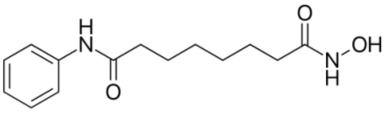
DB01162<br>Terazosin: an alpha-1 adrenergic antagonist used in the treatment of symptomatic benign prostatic hyperplasia	0.868832	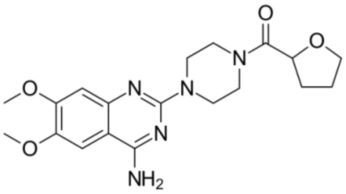
DB00590<br>Doxazosin: an alpha-1 adrenergic receptor used to treat mild to moderate hypertension and urinary obstruction due to benign prostatic hyperplasia	0.866061	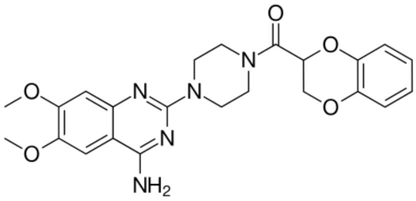
DB00346<br>Alfuzosin: an alpha-1 adrenergic antagonist used in the symptomatic management of benign prostatic hypertrophy	0.863308	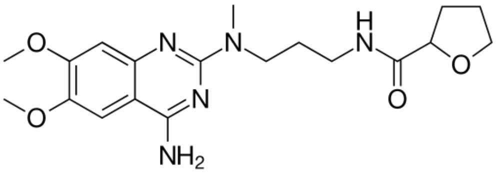
DB00316<br>Acetaminophen	0.860759	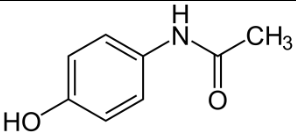
DB00461<br>Nabumetone: an NSAID used to treat osteoarthritis and rheumatoid arthritis	0.860696	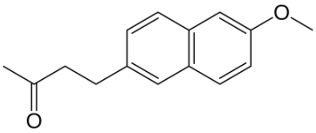

DB00750<br>Prilocaine: a local anesthetic used in dental procedures	0.860696	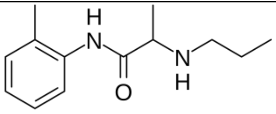
DB01113<br>Papaverine: an alkaloid used to treat many types of smooth muscle spasms	0.860696	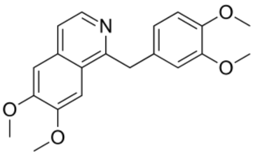
DB14895<br>Vibegron: a beta-3 adrenergic agonist the treatment of overactive bladder with symptoms of urge urinary incontinence	0.860572	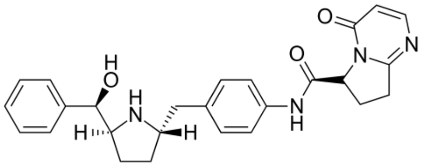
DB00304<br>Desogestrel: a synthetic progestin used in contraception, often in combination with ethinyl estradiol	0.859565	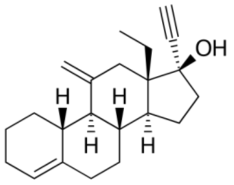
DB00243<br>Ranolazine: an anti-anginal drug used for the treatment of chronic angina	0.859565	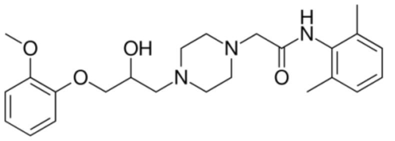
DB05294<br>Vandetanib: an antineoplastic kinase inhibitor used to treat symptomatic or progressive medullary thyroid cancer	0.857854	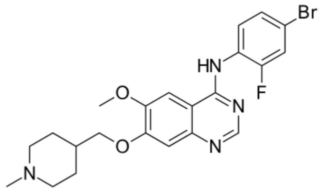
DB00268<br>Ropinirole: a non-ergoline dopamine agonist used to treat the symptoms of Parkinson's disease and Restless Legs Syndrome	0.857468	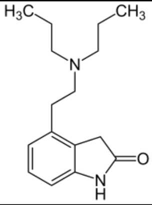
DB08893<br>Mirabegron: a beta-3 adrenergic agonist used to treat overactive bladder and neurogenic detrusor overactivity	0.85663	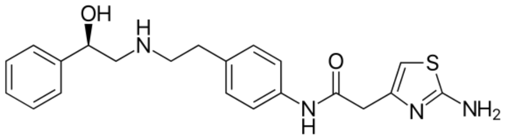
DB00317<br>Gefitinib: a tyrosine kinase inhibitor used as first-line therapy to treat non-small cell lung carcinoma	0.855152	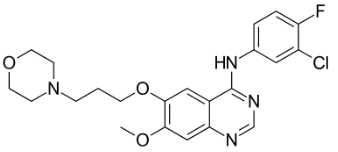
DB05676<br>Apremilast: a non-steroidal medication used for the treatment of inflammatory conditions such as psoriasis	0.855152	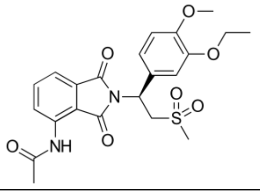

DB06594<br>Agomelatine: a potent agonist at melatonin receptors and an antagonist at serotonin-2C receptors	0.854264	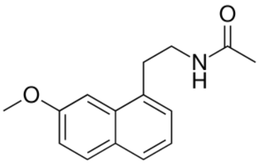
DB00457<br>Prazosin: an alpha-blocker that causes a decrease in total peripheral resistance and is used to treat hypertension	0.853716	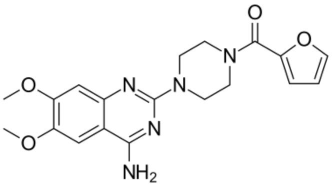
DB11800<br>Tivozanib: a kinase inhibitor to treat adult patients with renal cell carcinoma (RCC) who have failed prior systemic therapies or experienced relapsed disease	0.852468	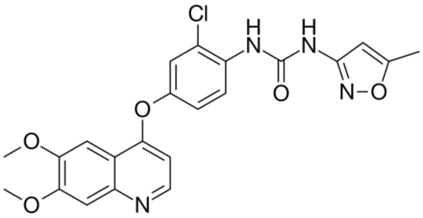
DB09089<br>Trimebutine: a spasmolytic agent used for the symptomatic treatment of irritable bowel syndrome (IBS) and treatment of postoperative paralytic ileus	0.851083	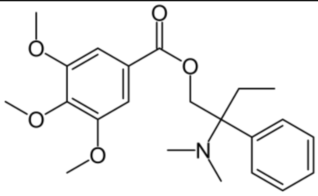

Table S2. The list of FDA-approved drugs with similarity index greater than 0.85 to G721-0282

Drug bank Info	Similarity index	Structure
DB00277<br>Theophylline: a xanthine used to manage the symptoms of asthma, COPD, and other lung conditions caused by reversible airflow obstruction	0.921921	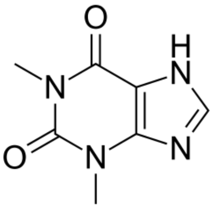
DB01223<br>Aminophylline: a bronchodilator consisting of theophylline that is used for the treatment of bronchospasm due to asthma, emphysema and chronic bronchitis	0.921921	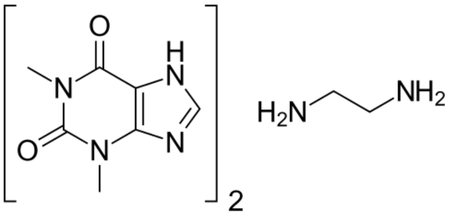
DB14018<br>Bromotheophylline: the active moiety of pamabrom, a mixture of 2-amino-2-methyl-propanol and bromotheophylline. From this mixture, bromotheophylline acts as a weak diuretic that has been used along with some analgesics to relieve the symptoms of premenstrual syndrome	0.90612	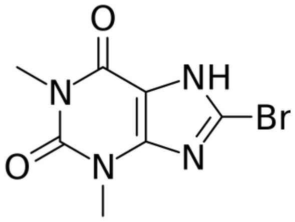
DB00651<br>Dyphylline: a theophylline derivative used to treat asthma, bronchospasm, and COPD	0.893219	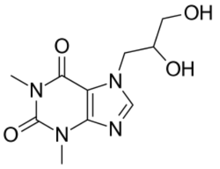
DB00812<br>Phenylbutazone: an NSAID used to treat backache and ankylosing spondylitis	0.887425	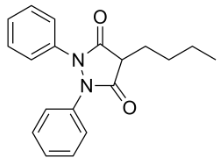
DB11254<br>Hexylresorcinol: an ingredient used to relieve irritation, pain, and prevent infection	0.875536	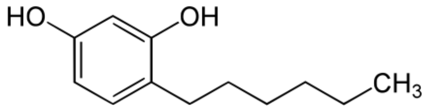
DB13908<br>Amylmetacresol: an antiseptic used to treat infections in the mouth and throat	0.875536	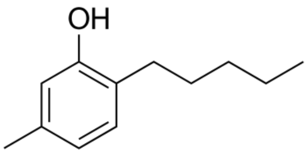

DB08868<br>Fingolimod: a sphingosine 1-phosphate receptor modulator used to treat patients with the relapsing-remitting form of multiple sclerosis	0.875536	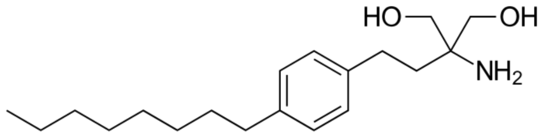
DB00806<br>Pentoxifylline: a methylxanthine derivative used to treat intermittent claudication caused by chronic occlusive arterial disease of the limbs	0.870529	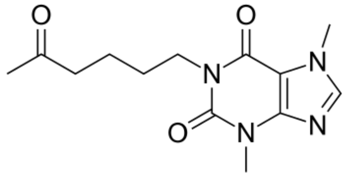
DB00824<br>Enprofylline: is a derivative of theophylline which shares bronchodilator properties	0.868085	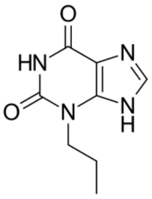
DB00418<br>Secobarbital: a barbiturate used for the short-term treatment of insomnia	0.868085	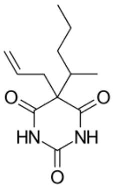
DB01154<br>Thiamylal: A barbiturate that is administered intravenously for the production of complete anesthesia of short duration, for the induction of general anesthesia, or for inducing a hypnotic state	0.868085	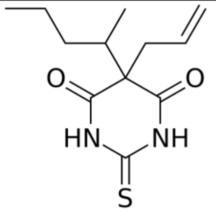
DB01353<br>Butobarbital: is a sedative and a hypnotic drug	0.854514	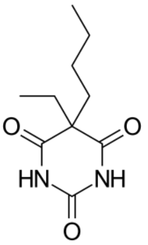
DB06203<br>Alogliptin: a dipeptidyl peptidase-4 (DPP-4) inhibitor used to treat hyperglycemia in patients with type 2 diabetes mellitus	0.854264	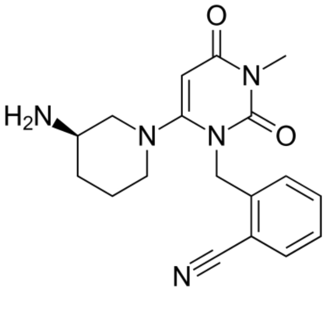

DB09061<br>Cannabidiol: an active cannabinoid used as an adjunctive treatment for the management of seizures associated with Lennox-Gastaut syndrome or Dravet syndrome and symptomatic relief of moderate to severe neuropathic pain	0.853556	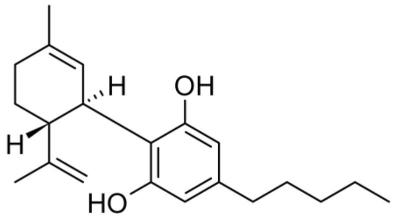
DB00312<br>Pentobarbital: a barbiturate drug used to induce sleep, cause sedation, and control certain types of seizures	0.85039	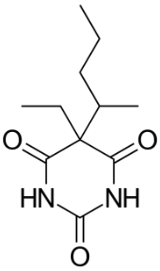
DB00599<br>Thiopental: a barbiturate used to induce general anesthesia, treat convulsions, and reduce intracranial pressure	0.85039	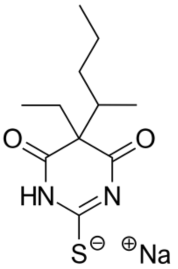

Figure S1. A) G721-0282 and B) K284's binding modes (rendered in magenta and green sticks, respectively) and detailed close-up of the ligand binding pocket, highlighting essential residues within a 5 Å proximity of the ligand as identified from MD simulations within the cavity (illustrated as a seafoam green cartoon), and two-dimensional diagrams of protein-G721-0282 and protein-K284 interactions. The yellow dashed lines denote hydrogen bonds, while the blue dashed lines represent π-π stacking.

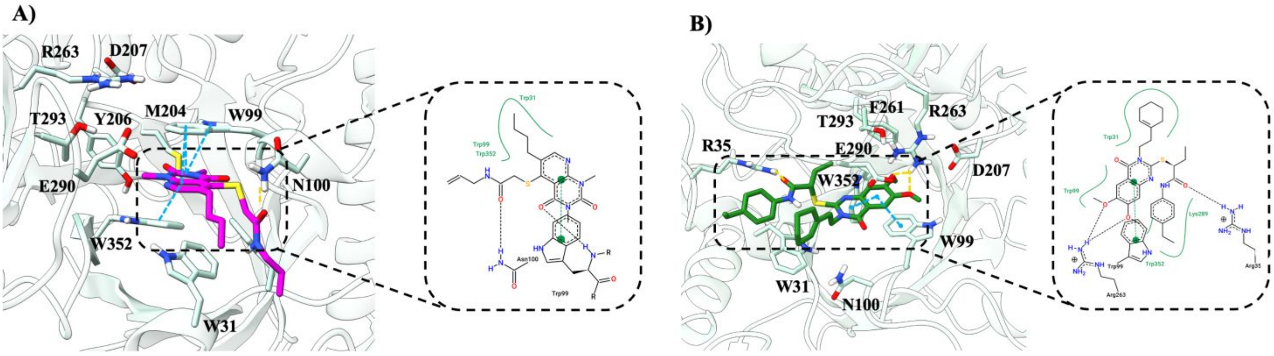

Figure S2. Two-dimensional diagrams of A) protein-fingolimod, B) protein-ponesimod, and C) protein-siponimod interactions. The black dashed lines denote hydrogen bonds and salt bridges, while the green dashed lines indicate π-π stacking.

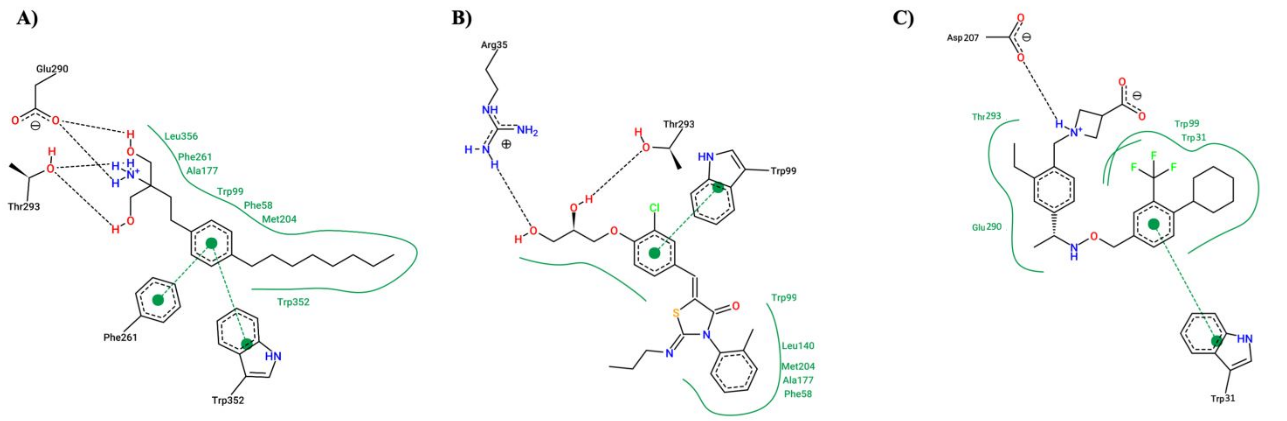

Figure S3. Essential dynamics analysis of the 500ns MD simulations run for CHI3L1- (A) fingolimod, (B) G721-0282, (C) K284, and D) ponesimod complexes. Only the first essential motion of the Cα atoms is shown.

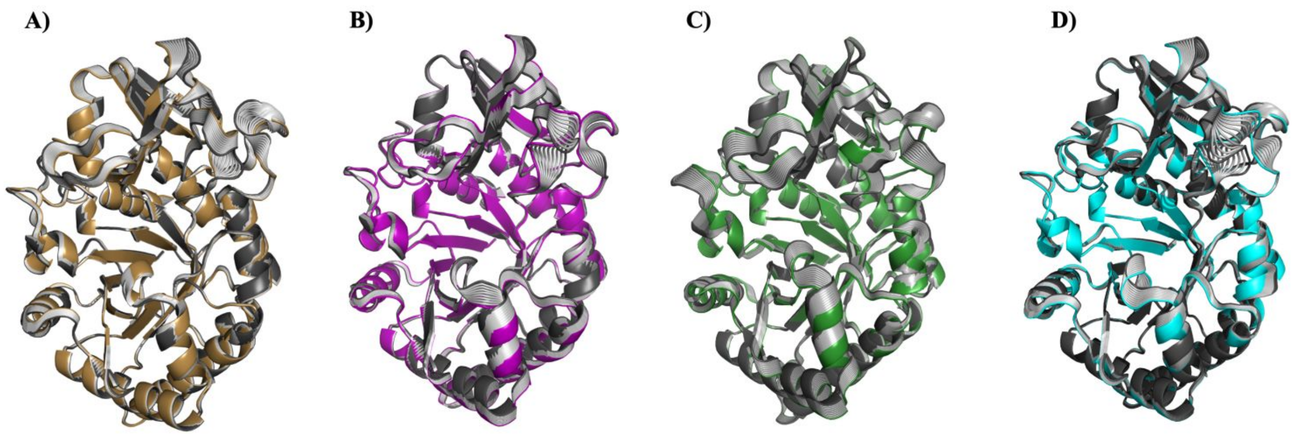

Figure S4. Superimposed binding poses of Inhibitor 30 (orange), G721-0282 (pink), fingolimod (purple) within the CHI3L1 binding pocket.

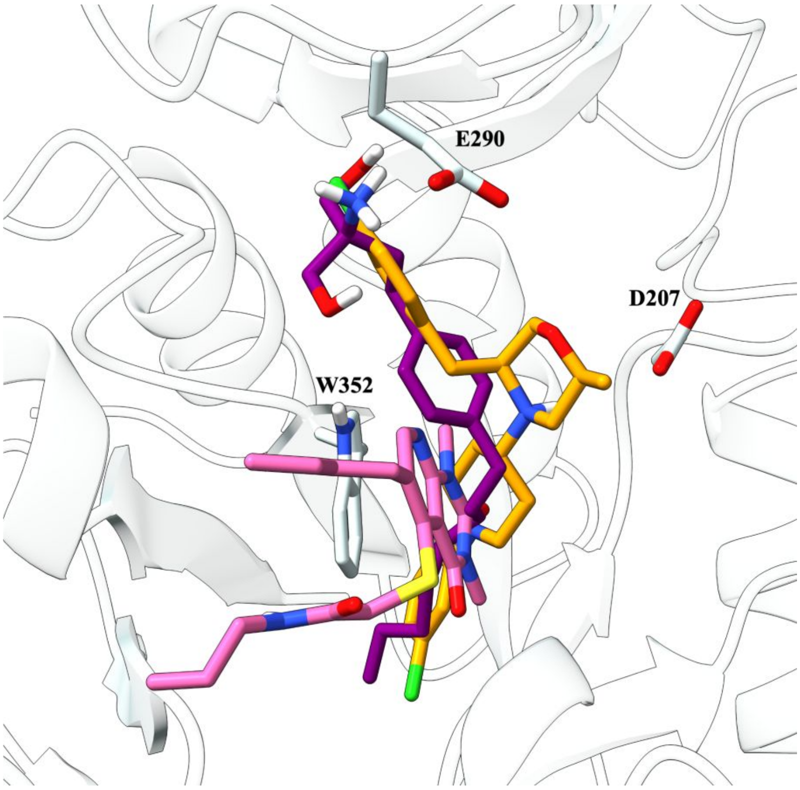

Table S3. Contribution of the essential motion (%) to the structural variance of different CHI3L1 complexes and the total contribution of the first three projections.

Complexes	*proj. 1*	*proj. 2*	*proj. 3*	*total$_{(P1-P3)}$*
inhibitor 30	7.7	6.2	4.5	18.4
fingolimod	12.5	8.6	5.3	26.4
G721-0282	14.7	8.0	5.3	28.0
K284	14.6	10.1	4.2	28.9
ponesimod	18.2	7.1	5.7	31.0
siponimod	19.0	7.0	5.8	31.8

SMILE of the compounds presented in the manuscript

Inhibitor 30:

ClC1=CC=NC(N2CCC(N3[C@@H](CC4=CC=C(Cl)C=C4)CO[C@@H](C)C3)CC2)=C1

Fingolimod:

CCCCCCCCC1=CC=C(CCC(N)(CO)CO)C=C1

Ponesimod:

CCC\N=C1/S\C(=C/C2=CC=C(OC[C@H](O)CO)C(Cl)=C2)C(=O)N1C1=CC=CC=C1C

Siponimod:

CCC1=CC(=CC=C1CN1CC(C1)C(O)=O)C(\C)=N\OCC1=CC=C(C2CCCCC2)C(=C1)C(F)(F)F

G721-0282:

O=C1C2=C(N=CC(CCCC)=C2SCC(NC=C)=O)N(C)C(N1C)=O

K284:

COC1=CC2=C(N=C(SC(C(NC3=CC=C(CC)C=C3)=O)CC)N(CCC4=CCCCC4)C2=O)C=C1OC
